# The Versatile Applications of DES and Their Influence on Oxidoreductase-Mediated Transformations

**DOI:** 10.3390/molecules24112190

**Published:** 2019-06-11

**Authors:** Fatima Zohra Ibn Majdoub Hassani, Saaid Amzazi, Iván Lavandera

**Affiliations:** 1 Biochemistry and Immunology Laboratory, Mohammed V University, Faculty of Sciences, BP 1014, Avenue Ibn Batouta Agdal, Rabat 10090, Morocco; amzazi@gmail.com; 2 Organic and Inorganic Chemistry Department, University of Oviedo, 33006 Oviedo, Spain

**Keywords:** deep eutectic solvents, biocatalysis, oxidoreductases, asymmetric synthesis

## Abstract

In the last decade, new types of solvents called deep eutectic solvents (DES) have been synthesized and commercialized. Among their main advantages, they can be eco-friendly and are easy to synthesize at different molar ratios depending on the desired solvent properties. This review aims to show the different uses of DES in some relevant biocatalytic redox reactions. Here we analyze oxidoreductase-mediated transformations that are performed in the presence of DES and compare them with the ones that avoided those solvents. DES were found to present advantages such as the increase in the product yield and enantiomeric excess in many reactions.

## 1. Introduction

Nowadays, industries seek to use novel types of solvents that can offer advantages over the more typical organic ones due to environmental reasons. Among them, the so-called deep eutectic solvents (DES) have recently appeared, which have great potential and can have many applications at an industrial scale, as they can be beneficial from a green chemistry point of view. DES are mixtures of a salt such as choline chloride (ChCl) and a hydrogen bond donor (HBD) molecule such as urea (Ur). These solvents present many advantages, including the facts that they are eco-friendly, cheap, and can increase the (bio)catalytic activity of many reactions leading to high yields of products with excellent enantiomeric excess (*ee*), including important precursors for drugs that are widely used in the pharmaceutical industry [1,2]. ILs are salts, consisting of a mixture of cations and anions that do not pack well together, that consequently have a melting point near room temperature, although they are arbitrarily defined as salts with a melting point below 100 °C [3]. The first example of an IL was described by Walden in 1914, when he obtained ethyl ammonium nitrate [4]. As a result, the so-called first generation of ILs appeared. However, this first generation suffered from some disadvantages, such as toxicity and molecular instability due to their high reactivity with oxygen. In 1992 Wilkes and Zaworotko [5] developed the second generation of ILs that were more stable against water and air. The anions were replaced with halides and those ILs had low melting points and were more stable in the presence of organic solvents and were used, among other applications, in biocatalysis in the 2000s. However, these ILs had again some disadvantages such as high cost and toxicity [2].

The reasons above led scientists to synthesize new eco-friendly ILs that could be used in, e.g., catalysis, and this type of ILs has been called DES. These solvents contain more stable anions and cations which are usually biodegradable. They are easy to prepare and do not require purification. In addition to that, enzymes have shown good activities and stabilities in DES [2]. This medium presents many potential advantages in the medical and biotechnological sectors. But before using them, one has to test their safety of use and the extent of their toxicity before they can be implemented for biotechnological applications, especially the ones involving the utilization of living cells. Mbous et al. [6] have summarized the toxicity of DES against different living organisms (Table 1).

In general, DES are composed of a hydrogen bond acceptor (HBA) and a HBD. According to Smith et al. [12] the general formula of a DES is made up of three components. The first component includes the cations which could be: choline, ammonium, sulfonium, or phosphonium cations. The second component of DES is a halide anion. The cations and halide anions make up the HBA part of the DES. The third component of the DES is a HBD, which includes a range of molecules such as: metal halides, amides, carboxylic acids, alcohols, urea, acetamide, or polyols. Based on the composition, they have been divided into four different classes. The first types are the ones composed of metal halides and imidazolium salts. They are composed of non-hydrated metal halides such as iron chloride. The second type of DES is composed of ChCl and hydrated metal halides such as copper chloride. These solvents are not sensitive to air and moisture. The third type of DES is formed by ChCl as a HBA and an amide, a carboxylic acid, or an alcohol, which act as a HBD. The fourth and last type of DES is made of a transition metal halide like zinc chloride, and with a HBD such as urea, acetamide, ethylene glycol, or hexane-1,6-diol [12].

Natural deep-eutectic solvents (NADES) can be considered as a type 3 DES because they are composed of a salt and a HBD molecule. However, one might argue that DES can be considered as green solvents only if they fulfill the standardized safety requirements compared to ILs and organic solvents. For this reason, a standardized protocol to control the quality and greenness of DES must be established in the near future [6].

It is important to mention that the properties of DES can be easily tuned by modifying their structures, by changing both the HBA and the HBD structures, and by adjusting the molar properties of both components. In a study conducted by D’Agostino and co-workers in 2015 [13] to characterize the DES in detail, they examined the composition of different DES and their relation with aqueous mixtures, showing the behavior of DES with water at different proportions. They also used pulsed field gradient nuclear magnetic resonance (PFG NMR) to determine active species in aqueous mixtures containing DES and to investigate their diffusion behaviors in those mixtures. DES composed by ChCl and glycerol, ethylene glycol, and urea were studied at a 1:2 molar ratio. The authors found that when the water content was increased in those three types of DES, the viscosity of those liquids decreased. Therefore, it was concluded that the fluidity of the liquid depended on the hydrogen bonds that were formed between the HBD and the halide anion. This study also gave new microscopic insights about complex liquid mixtures. NMR was used in this study to detect the mobility of charged and uncharged species.

According to Guajardo et al. [14], DES tend to perform better in reactions that contain a minimum amount of water. Also, these authors highlighted the different applications of DES and showed that they can be used as solvents in different catalytic reactions, such as metal-, organo- or biocatalyzed processes speeding up the reactions and enhancing the isolated product yields. In addition, DES can be used as convenient solvents to separate organic compounds like alcohols and esters from a complex reaction mixture.

Biocatalysis is the field of chemistry that focuses on the use of enzymes from living organisms to catalyze and speed-up the chemical reactions for synthetic purposes [15]. Regarding the biocatalysis field, Domínguez de María and Hollmann showed that the enzyme activity and selectivity strongly depend on the solvent properties [16]. Although biocatalysis is considered a green technology, the authors investigated how in some specific cases biocatalysis can affect negatively the environment. Sometimes, biocatalytic reactions involving water can yield by-products that are toxic to the environment. In these cases, the use of DES can afford a lower impact on the environment by extracting them. The best feature that enables different types of DES to dissolve enzymes and highly polar organic molecules is their capability to form hydrogen bonds.

This review is about the major effects and the latest applications of DES in oxidoreductase-mediated reactions and compares some applications of DES to other solvents that are widely used in this biocatalytic field. These enzymes are responsible of redox transformations and for this they require a cofactor, e.g., β-nicotinamide adenine dinucleotide, which exists in a phosphorylated (NADP^+^) and non-phosphorylated (NAD^+^) oxidised form, or in their reduced versions [NAD(P)H]. These cofactors can mediate as electron acceptors/donors in either oxidation or reduction processes. Since their use in stoichiometric amounts is hampered due to inhibition effects and economic hurdles, efficient cofactor recycling systems have been designed for the development of effective and economic feasible redox transformations [17].

## 2. Applications of Deep Eutectic Solvents to Oxidoreductase-Catalyzed Reactions

The first report related with the use of DES in the presence of an enzyme was the work of Kazlauskas and co-workers [18], who confirmed that lipases from different lyophilized bacterial cells had good catalytic activities in DES. It was also shown that other hydrolases had excellent activities in DES. That research group conducted several test reactions to observe the effect of DES on the lipase-catalyzed transesterification of ethyl valerate with butan-1-ol using eight different DES, and they compared their conversions with the ones obtained in toluene. It was found that DES improved significantly the biocatalytic reactions. Also, the initial specific activity of the lipases was higher in these media compared to the usual ILs that were previously used in these types of reactions. DES also enhanced the reaction rates up to 20-fold. The study also confirmed that they were convenient solvents in the case of the epoxide hydrolase-catalyzed transformation of styrene oxide regarding dimethylsulfoxide (DMSO) or acetonitrile.

Oxidoreductases are extremely important enzymes involved in aerobic and anaerobic metabolism in living organisms. As examples, some bioreactions that involve oxidoreductases include: Krebs cycle, glycolysis, amino acid metabolism and oxidative phosphorylation. Nowadays, oxidoreductases have gained attention in the biocatalysis field and have encountered industrial applications due to their ability to synthesize enantiopure compounds under mild conditions [19]. In this section, we will discuss the effect of DES on some oxidoreductase enzymes. In order to maintain or improve the activity of the enzyme similar to the one *in vivo*, scientists have used green solvents such as DES to help them transform lipophilic substrates more efficiently [20].

### 2.1. Alcohol Oxidase and Peroxidase-Catalyzed Transformations

**Scheme 1:**
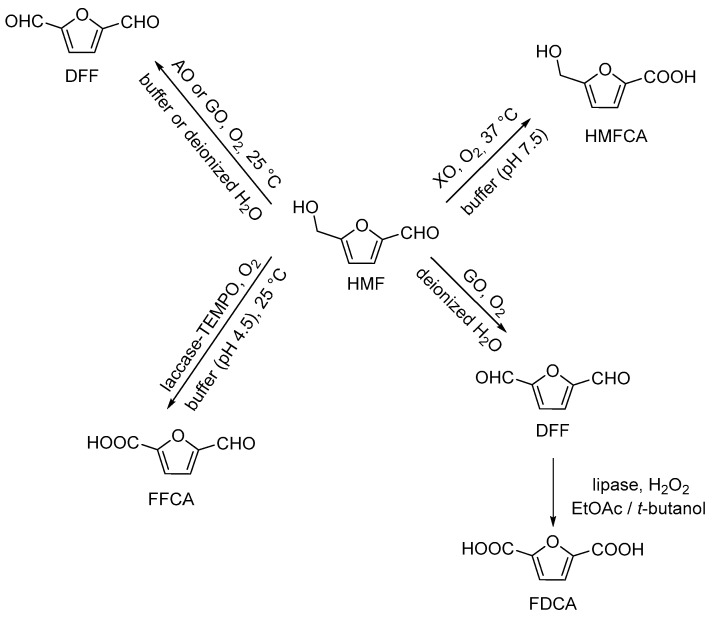
Enzymatic toolbox showing the oxidation of 5-hydroxymethylfurfural (HMF) to other valuable products [21].

A recent study has shown the use of different enzymatic preparations to selectively oxidize 5-hydroxymethylfurfural (HMF, Scheme 1), a natural product, to 2,5-diformylfuran (DFF), 5-hydroxymethyl-2-furancarboxylic acid (HMFCA), 5-formyl-2-furancarboxylic acid (FFCA), and 2,5-furandicarboxylic acid (FDCA) [21]. These oxidized derivatives are precursors of important compounds. For instance, DFF and FDCA have antifungal and anti-*Pneumocystis carinii* activities. Also, HMFCA is an important monomer for synthesizing polyesters. Alcohol oxidase (AO) from *Candida boidinii* oxidized HMF and showed a high activity compared to the other alcohol oxidases. AO was able to oxidize HMF to DFF in 41% yield. In addition, galactose oxidase (GO) from *Dactylium dendroides* was also able to perform this transformation in excellent yields (~90%) in the presence of catalase and horseradish peroxidase. Xanthine oxidase (XO) from *Escherichia coli* was able to selectively oxidize the formyl group located in the HMF substrate and gave HMFCA in 94% yield after only seven hours (Scheme 1). In addition, this XO-mediated process had many advantages, as it used air instead of the toxic H_2_O_2_ to achieve the oxidation, it did not use harmful organic solvents, it was highly selective, and therefore, no by-products were observed [21].

This research team also used three different laccases applied to the oxidation of HMF to FFCA in the presence of the mediator 2,2,6,6-tetramethyl-1-piperidinyloxy radical (TEMPO). At the beginning of the reaction, DFF appeared as an intermediate and was then transformed to FFCA that was accumulated along the time. The laccase from *Panus conchatus* gave the highest yield of FFCA (82%) after 96 h. The laccase from *Trametes versicolor* gave a 68% yield of FFCA after 48 h, and the laccase from *Flammulina velutipes* afforded a 70% yield of FFCA after 72 h. For the synthesis of FDCA, a sequential oxidation protocol was achieved, combining GO and lipase from *Candida antarctica* type B (CAL-B). In a first step, GO oxidized HMF to DFF (75% conversion) as previously described after 48 h. After extraction with ethyl acetate (EtOAc), *t*-butanol, CAL-B and H_2_O_2_ were added to the reaction medium to convert DFF to FDCA. An excellent yield of FDCA in this second step was obtained after 24 h (88%).

In the same study, the successful separation of HMF from DFF with the help of DES was also described after the enzymatic transformations, because DES have affinity towards hydrogen bond-containing molecules, and they enable to dissolve those molecules. The authors were able to separate DFF from HMF using three types of DES. The composition of these solvents was: choline chloride and glycerol (ChCl:Gly 1:2 mol/mol), choline chloride and urea (ChCl:Ur 1:2 mol/mol), and choline chloride and xylitol (ChCl:Xyl 1:1 mol/mol). They were used to extract HMF from an ethyl acetate solution that contained both DFF and HMF. The researchers discovered that DES composed of ChCl:Gly and ChCl:Ur were the ones that selectively extracted HMF from the mixture. When this mixture was extracted three-times using ChCl:Gly DES, the purity of DFF increased to 97% from the original purity of 76%.

**Figure 1:**
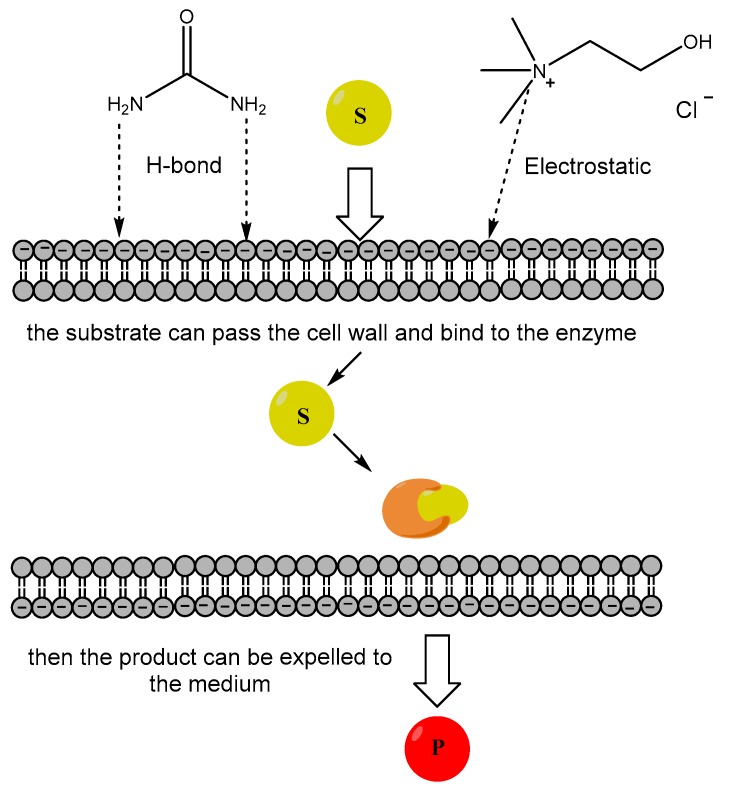
The effect of deep eutectic solvents (DES) on cell membrane permeability. The cations from the choline chloride and urea that form hydrogen bonds can disrupt the cell membrane, allowing the substrate to enter the cells, so then it can bind to the appropriate enzyme. Finally, the product can be released to the medium.

In another investigation conducted by Yang et al., they tested the effect of 24 DES and 21 NADES as co-solvents on the biotransformation of isoeugenol into vanillin using *Lysinibacillus fusiformis* CGMCC1347 whole cells. It was discovered that those eutectic solvents (1% v/v) enhanced the bioconversion because it made the bacterial cell membrane permeable to the lipophilic substrate (Figure 1). Among them, the DES composed of choline acetate (ChAc) were usually better than the ones composed of ChCl in terms of product yields (up to 142%) regarding the control reaction without DES [22]. The whole cells were immobilized on poly(vinyl alcohol)-alginate beads, and this biotransformation could be repeated up to 13 times in the presence of choline chloride:galactose (ChCl:Gal 5:2 mol/mol, 20% v/v), maintaining their activity to a similar extent, offering this system a promising design for further developments.

In a study conducted by Wu et al. [23], it was found that DES composed of ChCl and different HBDs (Ur, Gly, acetamide, and EG) at different molar ratios promoted more efficiently the activity of horseradish peroxidase (HRP) compared to DES which was composed of choline acetate. In that study, twenty-four DES were synthesized to see their effect on HRP activity, and they were found to have a stabilizing effect on HRP, especially those when the molar ratio of the salt was higher than that of the HBD. The authors also identified through spectroscopic studies that DES were able to enhance the α-helix conformations thus providing a more relaxed tertiary structure of the enzyme, hence improving its activity. They concluded that DES can be versatile solvents and found that the hydrogen bonding network that is provided by DES prevents this solvent from dissociation in aqueous solutions at least at concentrations of 0.5 M.

Luna-Bárcenas and co-workers in 2016 have used two types of DES to conduct an enzyme-mediated free radical polymerization of acrylamide. One DES was made of ChCl:Ur and the other was made of ChCl:Gly (1:2 molar ratio). When the DES was used at higher volumes than the phosphate buffer solution, the catalytic activity of the HRP diminished drastically. However, the thermal stability of the enzyme was improved. The enzyme was still able to achieve the initiation of the acrylamide free radical polymerization even at 80% v/v concentrations of DES. Also, the research group was able to synthesize polyacrylamide in ChCl:Gly at 4 °C because it has a low freezing point. The combination of HRP, H_2_O_2_ and pentane-2,4-dione as initiator was responsible to promote the reaction leading to polyacrylamide synthesis in aqueous medium. The free radical polymerization occurred also at room temperature and at 50 °C. HRP was partially denatured in ChCl:Ur because the heme group was extracted into the solution and this caused a decrease in the enzymatic activity at higher DES concentrations. The concentrations of ChCl:Ur and ChCl:Gly were fixed to 80% v/v when used in the reaction media for acrylamide polymerization to protect the hydrogen bonding network in the hydrated medium while decreasing the viscosity of these solvents. Also, a homogenous mixture was obtained throughout the process [24].

Another study conducted by Papadopolou et al. [25] investigated the effect of DES made of ChCl or ethylammonium chloride (EAC) and different HBDs such as Ur or Gly at different molar ratios, on the catalytic activities of cytochrome c (cyt c) and HRP. This research team showed that the activity of the peroxidase depended on the nature of both the ammonium salt and the HBD. In addition to that, the study confirmed that EAC-derived DES stabilized cyt c and enhanced its capabilities for the degradation of an industrial dye compared to the eutectic mixture containing ChCl, suggesting that these eco-friendly solvents could be an interesting medium for biocatalytic reactions with applications in industry. When they compared the peroxidase activity in pure buffer and in DES-based media, it was concluded that the neoteric media increased the activity of the enzyme. In the case of choline chloride-based DES, when ChCl:Ur was used in the reaction medium at a concentration of 30% v/v, the activity of cyt c was enhanced up to 8-fold times compared to the one in buffer, while using EAC:Ur at 50% v/v it was possible to increase its activity up to 200-fold times. An explanation for this effect could be the higher viscosity of ChCl-based DES, increasing the mass transfer limitations of the substrates. Also, the HBD affected the activity of cyt c. When Ur was used, its activity was largely increased compared to the ones observed for DES composed of Gly and EG. The thermostability of cyt c could also be enhanced using DES as reaction media. Hence, when the enzyme was incubated with buffer or in the presence of 30% v/v of different DES, while cyt c lost 35% of its activity after incubation in buffer at 40 °C after 24 h, it remained perfectly stable when it was incubated in ChCl:Ur or EAC:Ur media. As an application, the decolorization of a dye, pinacyanol chloride, in different DES was tested using cyt c. The authors could immobilize the enzyme on Celite and perform the decolorization of this compound up to four cycles observing excellent activities using EAC:Ur and EAC:EG at 50% v/v [25].

### 2.2. Deep Eutectic Solvents and Alcohol Dehydrogenases (ADHs)

ADHs are probably the most used enzymes corresponding to the family of oxidoreductases for synthetic purposes. They catalyze the reversible transformation between alcohols and carbonyl compounds through redox processes. These reactions are mediated by a nicotinamide cofactor, namely NAD or NADP, which is responsible of the electron transfer from or to the substrate [26]. Apart from using free, isolated enzymes, the setup of whole cell-mediated transformations enables simple and cheaper procedures. Especially interesting is their use applied to the cofactor-dependent reactions, due to the fact that whole cell microorganisms can provide it on its own and avoid the external addition of these expensive molecules. As eco-friendly and cheap solvents, DES have been selected as good solvent candidates for ADH-mediated protocols. As the subsequent studies will show, DES present many stabilizing effects on biocatalytic reactions involving ADHs from different organisms.

ADHs are extremely important enzymes due to many reasons. For instance, in the human body they are the main enzymes responsible for digesting and degrading the alcohol that is consumed. These enzymes are usually present in the human liver and they detoxify ethanol converting it into acetate that is later used by cells. Moreover, ADHs from yeast are used to convert glucose to ethanol to make alcoholic beverages [27]. Also, from a synthetic point of view, ADHs from microorganisms are used to convert ketones into chiral alcohols that can be precursors and building blocks of some important drugs such as cholesterol lowering drugs, anti-arthritic, and antibacterial agents. It is worthy to mention that these enzymes reduce ketones selectively according to the Prelog’s rule, which can be used to predict the stereopreference of alcohol dehydrogenase-catalyzed carbonyl reductions. Hence, ADHs usually provide the hydride through the *Re* face of this prochiral moiety. In that case, the ADH follows the Prelog’s rule; on the contrary, the biocatalyst will show anti-Prelog selectivity [28].

**Scheme 2:**
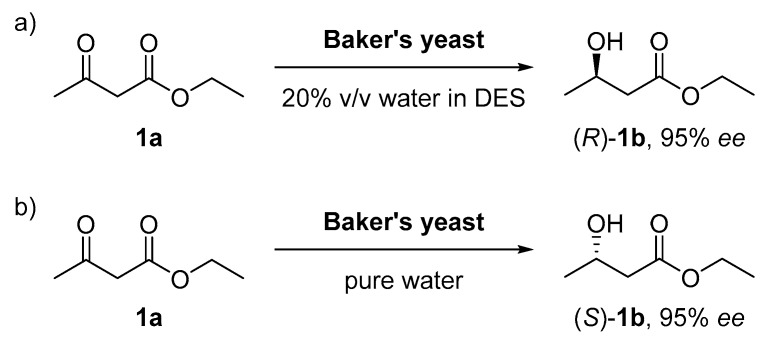
The stereoselectivity inversion in the formation of alcohol **1b** based on the type of solvent used. (**a**) Baker’s yeast cells showed a high stereoselectivity towards the (*R*) enantiomer when some amount of water was mixed with the DES (choline chloride:glycerol 1:2 mol/mol). (**b**) A high (*S*)-enantioselectivity was obtained when pure water was used with that biocatalyst.

As a first example, Domínguez de María and co-workers demonstrated that DES could be an appropriate media for whole cell catalysis, achieving the bioreduction of ethyl acetoacetate (**1a**) with Baker’s yeast [29]. This study showed that DES can be efficient inhibiting oxidoreductases. For instance, alcohol dehydrogenases with (*S*)-stereopreference were inhibited by ChCl:Gly (1:2 mol/mol). When adding different proportions of this DES into water, it was observed the inversion of the stereoselectivity in the bioreduction of **1a** using this biocatalyst. Baker’s yeast whole cells showed an excellent stereoselectivity towards the (*S*)-enantiomer of **1b** in pure water (95% *ee*). However, when the reaction was left in a DES medium containing less than 20% volume of water, the product was obtained with high (*R*)-enantioselectivity (95% *ee*, Scheme 2). The reason for that was because Baker’s yeast genome contains a mixture of approximately 50 ADHs that can present different selectivities. Therefore, this effect could be explained due to the fact that some (*S*)-selective enzymes were inhibited by DES while (*R*)-selective ones were more active in this medium. In conclusion, DES were proven to be great solvents for whole cell biocatalysis, being able to influence both activity and selectivity. 

In 2015 Bubalo et al. [30], tested different DES on yeast mediated reduction of **1a** and found that the DES that were composed of a sugar derivative had the best biocompatible results. Also, it was found that the amount of water present in the DES influenced the reaction results leading to an increase in the reaction yield when enlarging the percentage of DES. That group tested the effect of different types of DES using the yeast *Saccharomyces cerevisiae* (baker’s yeast). They found that the reaction yield was influenced by the type of HBD (e.g., glucose, fructose, or glycerol) and by the amount of water added to the prepared DES solution. It was concluded that the aqueous solutions of DES (50% v/v of water) gave the highest reaction yield (93%). The viability of yeast cells in DES containing ChCl and Ur was also investigated, and it was found that it decreased in this type of solvent. The increased osmotic pressure on yeast cells grown in the urea type of DES led to a decrease in the viability of the cells. As a result, water diffused out of those cells. However, DES containing a sugar or glycerol demonstrated a yeast viability of 76–99% after 24 h of inoculation of the yeast cells. DES containing a sugar or glycerol provided a pH around 4.5, while the DES containing urea afforded a pH superior to 8. Since the preferred pH range for *S. cerevisiae* growth is from 4 until 6, and as mentioned before the pH for DES containing sugars was 4.5, the best bioreduction yields were observed in these media. These results confirm that pH values and cell viability in bioreduction reactions involving DES can be an important effect, strongly influencing the enzymatic results. Glucose and other sugars such as fructose can also play an important role in the cofactor regeneration necessary for these processes [30].

**Figure 2:**
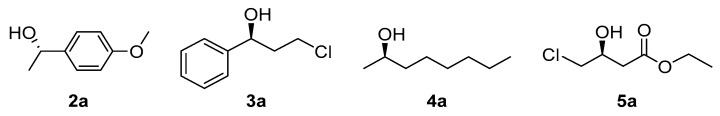
Examples of chiral alcohols that were obtained in alcohol dehydrogenase (ADH)-biocatalyzed processes in the presence of DES.

In a subsequent study done by Xu et al. [31], the authors used different DES to study the enantioselective oxidation of racemic 1-(4-methoxyphenyl)ethanol (MOPE, **2a**) employing whole cells of *Acetobacter* sp. CCTCC M209061 and acetone to recycle the oxidized nicotinamide cofactor. They found out that the DES composed of ChCl:Gly (1:2 mol/mol) at 10% (v/v) afforded the best results because it improved the permeability of the cells membrane. It also improved the stability of the enzyme(s) involved in the process. Therefore, it was possible to obtain the remaining (*S*)-**2a** (Figure 2) at 50% conversion in enantiopure form at a substrate concentration of 55 mM, higher than the one used in plain buffer (30 mM). Enantiopure (*S*)-MOPE can be used to make cycloalkyl[*b*]indoles which treat allergic responses.

In another study developed by the same group, it was demonstrated that DES ChCl:Ur (1:2 mol/mol) was able to improve the asymmetric reduction of 3-chloropropiophenone to (*S*)-3-chloro-1-phenylpropan-1-ol (**3a**, Figure 2) catalyzed by *Acetobacter* sp. CCTCC M209061 whole cells immobilized on PVA-sodium sulfate using glucose to recycle the cofactor [32]. Among the different DES studied, ChCl:Ur increased the permeability of the bacterial cells as it was confirmed by flow cytometry. By augmenting it, the biocatalyst(s) could bind better the ketone substrate to convert it into the corresponding enantiopure alcohol. Hence, using 5% v/v of DES at 10 mM substrate concentration, the product yield was 82.3% and the *ee* >99%. Later, utilizing a mixture of this DES with a water-immiscible ionic liquid (1-butyl-3-methylimidazolium hexafluorophosphate) in a biphasic system, it was possible to further increase the productivity of this transformation to 1.87 mmol/L.h.

In 2015, Müller et al. tested the effect of DES on the activity and stereoselectivity of ADHs from *Ralstonia* sp. (RasADH), *Thermoanaerobacter ethanolicus* (TeSADH) and horse liver (HLADH), which were overexpressed on *E. coli*. They used aliphatic and aromatic ketones as substrates and different DES proportions. It was found that these solvents could enhance the selectivity of the ADHs, maintaining a good activity even at high DES concentrations. In addition, the best solvent was the mixture ChCl:Gly (1:2 mol/mol) and buffer at a proportion of 80:20 v/v. The three ADHs that were used showed excellent activities when different DES-buffer proportions were utilized with the substrates: octan-2-one (for TeSADH), benzaldehyde (for HLADH), and propiophenone (for RasADH). In another set of experiments, the performance of RasADH with propiophenone as a substrate was tested. When the ancillary cosubstrates (ethanol, propan-1-ol, and propan-2-ol) were tested in the DES-aqueous-media mixtures, conversion remained high (>80%) and *ee* towards the (*S*)-alcohol were enhanced up to >95%. For other substrates a similar trend was observed, obtaining high *ee* for some aromatic substrates [33].

A research study led by Xu et al. explored different DES compositions and their effects in the reduction of octan-2-one using whole cells of *Acetobacter pasteurianus* GIM1.158 to obtain (*R*)-octan-2-ol (**4a**, Figure 2). They confirmed that DES composed of ChCl:EG (1:2 mol/mol) offered the best results in this transformation. Moreover, when combining this DES with an imidazolium based water-immiscible IL, the productivity of this bioreduction could be highly improved. Thus, using a mixture of ChCl:EG (32% v/v) and 1-butyl-3-methylimidazolium hexafluorophosphate (C_4_MIM•PF_6_, 20% v/v), in the presence of propan-2-ol as a hydrogen donor, 2-octanone (1.5 M) could be reduced at 90% conversion providing the enantiopure (*R*)-alcohol. Authors explained that DES increased the cell membrane permeability and kept the cells stable in the reaction system. In the case of the biphasic system, those solvents improved the substrate consumption by the cells because the second phase acted as a substrate reservoir, diminishing substrate or product inhibition and leading to a good yield of the desired product (*R*)-octan-2-ol [34].

Ethyl (*S*)-4-chloro-3-hydroxybutanoate ((*S*)-CHBE, **5a**, Figure 2) is known to be a precursor for drugs like atorvastatin calcium. In a study conducted by Dai and co-workers [35], (*S*)-CHBE was produced from the corresponding ketone using recombinant whole cells of *E. coli* CCZU-T15. Thus, when using ChCl:Gly (1:2 mol/mol) at 12.5% v/v in the presence of surfactant Tween-80 and L-glutamine, the substrate could be efficiently transformed (>93%) into the enantiopure alcohol at very high substrate concentrations (>2 M). L-Glutamine participated in the biosynthesis of the nicotinamide cofactors and could promote the biocatalytic activity, so no external addition of NAD was necessary. Another reason behind the high production of the product (*S*)-**5a**, was that the solvents and the surfactant increased the membrane permeability of the cells helping the substrate to bind to the enzyme(s) involved. This study confirmed that DES containing glycerol were appropriate solvents for the bioconversions that used recombinant bacterial whole cells.

A study performed by Panic et al. used NADES as solvents for plant cells biocatalysis. They were able to reduce 1-(3,4-dimethylphenyl)ethanone to the corresponding enantioenriched alcohol using carrot root. In addition, the hydrolysis of (±)-1-phenylethyl acetate by carrot root was also achieved in a eutectic mixture based on ChCl. Interestingly, although conversions were lower than in plain water, inversion of the stereoselectivity was obtained when increasing the percentage of DES. The NADES that were used in this study were made at different molar ratios of choline chloride and glucose, xylose, xylitol, glycerol, or ethylene glycol. In addition, the pH of the water solutions varied from 3.3 to 7.1. Different selectivities were obtained when the water content was changed in the NADES. Thus, usually when employing percentages of 30% v/v of the eutectic solvent made of choline chloride and glucose, the (*R*)-enantioenriched alcohol was obtained, while when increasing it up to 80% v/v, the (*S*)-antipode was predominantly observed. The reactions were made using up to 80 mM substrate concentration. This is another example that shows how the use of DES in combination with multienzymatic systems can greatly improve the selectivity observed in conventional media [36].

In 2016 Wei et al. were able to selectively oxidize racemic **2a** (MOPE) with *Acetobacter* sp. CCTCC M209061 cells more efficiently by the addition of DES in a two-phase system. In this research, the authors used two different systems in the biocatalytic asymmetric oxidation of MOPE to get (*S*)-**2a**. Water-immiscible organic solvents and ILs such as 1-butyl-3-methylimidazolium hexafluorophosphate were used as a second phase. Also, they added the DES ChCl:Gly (1:2 mol/mol) to the biphasic system to make the biocatalytic reaction more efficient. When the biocatalytic reaction occurred in the C_4_MIM•PF_6_/buffer medium, a substrate concentration of 65 mM could be converted at the maximum theoretical conversion value of 50.5%, affording the remaining enantiomer with an *ee* value of >99% after 10 h. When ChCl:Gly (10% v/v) was added to the aqueous phase, this enhanced the biocatalytic oxidation rate of the substrate. Hence, the initial reaction rate increased to 124.0 µmol/min, and the reaction occurred successfully only after 7 h with 51.3% conversion at 80 mM substrate concentration. As it was described, when the concentration of **2a** was enhanced, the initial rate increased continuously until having a substrate concentration of 80 mM. The highest initial reaction rate was attributed to the improved effect that DES had on the cell membrane permeability. The immobilized bacterial cells were able to keep 72% of their initial activity even after 9 batches of reuse in the C_4_MIM•PF_6_/ChCl:Gly‒buffer system. The researchers also used different systems to explore the transformation of MOPE to (*S*)-**2a** using the *Acetobacter* sp. cells in a 500-mL preparative scale. The asymmetric oxidation of the racemic substrate in the presence of DES was better compared to the one that occurred in the aqueous buffer-IL system, obtaining the desired alcohol in an enantiopure form [37].

In another investigation performed by Vitale et al., they explored the ability of baker’s yeast to reduce a series of ketones (Table 2) using DES as main solvents [38]. In those experiments, different eutectic mixtures were studied but the best results were obtained with the one composed of ChCl:Gly (1:2 mol/mol). The researchers proved that the amount of water and DES which were added to the reaction mixture was very important even for reversing the selectivity. Hence, the *anti*-Prelog reduction of different ketones could be achieved when ChCl:Gly DES was used with the whole cell biocatalyst due to the inhibition of the (*S*)-oxidoreductases present in it. In the case of phenylacetone (**6a**), the corresponding alcohol was produced with a high stereoselectivity in pure water, giving (*S*)-**6b** in 96% *ee*, while in DES‒aqueous mixtures up to 90% w/w of DES, the formation of the *R*-enantiomer was clearly favored (up to 96% *ee*). When water was used at 40% w/w, a racemic mixture of the alcohol was observed and there was no bioconversion when pure DES was employed in the reaction mixture. This team also proved that the outcome of the results depended on the composition of DES. The final stereoselectivity was obtained as a result of an interplay of whole cells “solvation” and the selective “inhibition” of some enzymes based on the nature of DES components. The DES that contained ChCl:glucose (2:1 mol/mol) and ChCl:Ur (1:2 mol/mol) deactivated the biocatalyst when used with 10% w/w water. Also, when water was replaced by ChCl:D-fructose DES mixture (3:2 w/w), this did not result in the conversion of phenylacetone to 1-phenylpropan-2-ol after five days. However, when this eutectic solvent was used with 40% w/w water, (*S*)-1-phenylpropan-2-ol was obtained with an *ee* value of 78% and the conversion was 31% after five days. This proved that the Baker’s yeast cells required a certain amount of water in order to show activity. It must be mentioned that the chiral (*S*)- and (*R*)-1-phenylpropan-2-ol (**6b**) derivatives are used in the preparation of neuroprotective drugs.

When the aqueous‒ChCl:Gly eutectic mixtures (10:90 w/w) were used with arylpropanone derivatives bearing electron-withdrawing groups (F or CF_3_), the chiral enantioenriched *R*-configured alcohols were obtained (> 40% *ee*) although at low extent. Thus, when 1-(4-fluorophenyl)propan-2-one (**7a**) was incubated at 37°C in water with Baker’s yeast, this substrate was reduced to (*S*)-**7b** with 82% conversion in 94% *ee*. However, the (*R*)-enantiomer was preferentially attained when this substrate was dissolved in a DES‒water (80:20 w/w) mixture in 14% conversion and 82% *ee*. When the amount of water was reduced to 10% w/w, the *ee* of (*R*)-**7b** increased to 90% and the conversion was 10%. Also, 1-(4-(trifluoromethyl)phenyl)propan-2-one (**8a**) was reduced to (*S*)-**8b** with very high conversion (90%) and *ee* (98%) in plain water while to (*R*)-**8b** (15% conv. and 40% *ee*) when using a mixture ChCl:Gly/water 90:10 w/w. With 1,1,1-trifluoro-3-phenylpropan-2-one (**9a**), a racemic mixture of the alcohol **9b** was achieved in water and the *ee* increased to 24% to form the (*R*)-enantiomer with a 12% yield utilizing the same DES at 90% w/w. When an additional methylene group existed between the aryl and the carbonyl group, the stereopreference did not change in the eutectic mixtures, obtaining the corresponding *S*-enantiomers as in the aqueous medium but with lower conversions and selectivities. Unfortunately, an aromatic and a heteroaromatic ketone were slightly or not reduced using baker’s yeast in the absence or presence of DES [38].

**Scheme 3:**
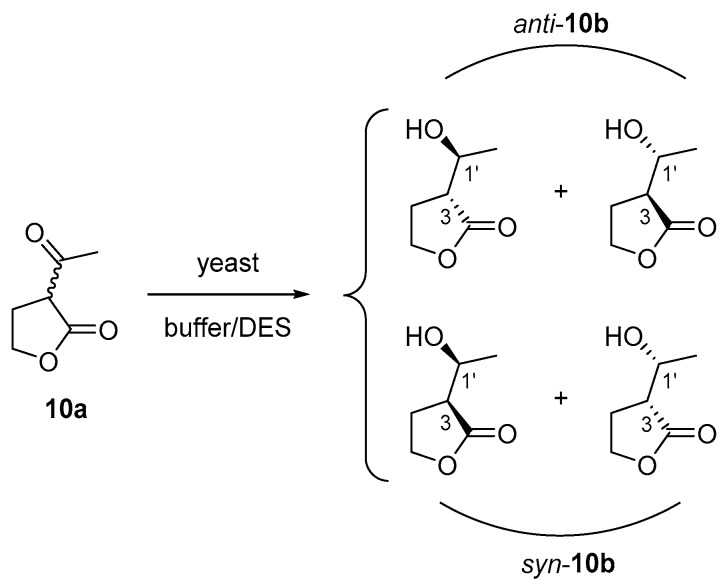
The possible diastereoisomers that can be obtained from yeast bioreduction of ketone **10a**.

In a study conducted by Maczka et al. they used seven yeast strains to transform 3-acetyldihydrofuran-2(3*H*)-one (**10a**) and get access preferentially to the *anti*-stereoisomers of the corresponding alcohol **10b** (Scheme 3) through a dynamic kinetic resolution. The authors also investigated the effect of the medium components on the efficiency and stereoselectivity of this biotransformation. Different strains of *Yarrowia* reduced successfully **10a** into *anti*-**10b**. Particularly, two strains of *Y. lipolytica* performed this transformation with very high *ee*. Hence, after one day, optically pure *anti*-(3*S*,1’*R*)-**10b** was produced using *Y. lipolytica* P26A in a medium composed of yeast extract (1% w/w), peptone (2% w/w), and glucose (2% w/w). On the other hand, *Candida viswanathi* AM120 was able to transform **10a** to a mixture of two isomers: *anti*-(3*R*,1’*S*)- and (3*S*,1’*R*)-**10b** with a trace amount of the *syn*-(3*S*,1’*S*)-enantiomer. Then, it was decided to add small quantities of different organic solvents to study their influence on the reductions mediated by the both yeast strains after 1 day. Thus, ethanol, glycerol, hexane and isopropanol were added to the medium (5-20% v/v) to enhance the solubility of the substrate. The alcohols could also regenerate the nicotinamide cofactors NADH or NADPH needed by the ADHs. In the case *C. viswanathi*, a reduction in the enantiomeric excess of the *anti*-(3*R*,1’*S*)-**10b** diastereoisomer was usually observed. Also, a large decrease in the conversion of the product was seen when ethanol was added to the reaction with *Y. lipolytica* P26A. In addition, the *syn* isomers were also detected when large proportions of the organic solvent were added. The use of glycerol or hexane up to 10% v/v did not have an effect on the reaction. Due to the good results achieved with glycerol, the authors confirmed that when adding a DES composed of ChCl:Gly (1:2 mol/mol), the substrate could be completely reduced after seven days with *C. viswanathi* AM120 cells in a buffer with a pH of 7. The DES also increased the stereoselectivity in the transformation. The addition of 10% v/v of DES on the resting cells resulted in the formation of *anti*-(3*R*,1’*S*)-**10b** with 85% diastereomeric excess and the *ee* was 76%, higher than in the previous cases in the absence of any additive or in the presence of other organic solvents. The researchers tried other strains such as *Hansenula anomala* C2 and *Saccharomyces pombe* C1, but they observed negligible or very low conversions [39].

Cicco et al. performed a research, where the asymmetric bioreduction of a series of ketones was achieved by using purified ketoreductases in the presence of DES as a part of a cascade protocol. These enzymes showed excellent catalytic performances and stabilities in mixtures made of DES and aqueous buffer. The most successful bioreductions occurred in the media containing ChCl:Gly (1:2 mol/mol)-buffer and ChCl:sorbitol (1:1 mol/mol)-buffer mixtures. It was demonstrated that when there was a high amount of DES in the mixture, a better enantioselectivity towards the formation of the secondary alcohol was obtained. Based on these results, they suggested that the DES-buffer mixtures could be used in a chemoenzymatic cascade process and could be run in both sequential and in concurrent modes: first the isomerization of racemic allylic alcohols, which is catalyzed by a ruthenium catalyst to afford the saturated ketone, and then this step was coupled with an asymmetric enzymatic reduction (Table 3). Thus, a racemic mixture of the allylic alcohol was converted to an enantiopure saturated secondary alcohol (*R* or *S*) without using isolation and purification steps of the ketone intermediate [40].

As a first step, it was explored the bioreduction of propiophenone to 1-phenylpropan-1-ol in different DES-buffer mixtures from 50 to 80% (w/w) using a set of ten commercial KREDs. The four different DES were composed of ChCl and Gly, Ur or lactic acid at a molar ratio of 1:2, and sorbitol at a molar ratio 1:1. Most of the KREDs used gave poor conversions in the presence of only ChCl, and these enzymes were inactive in the DES composed of Ur or lactic acid even at 50% w/w DES. However, the DES that contained sorbitol or Gly as a HBD and ChCl as a HBA allowed better conversions at 50% w/w. More than half of KREDs were still active in the presence of 80% w/w of both DES, staying especially very active in ChCl:Sorb (conv. >80%), particularly KRED-P2-C11. Since it is known that 16 out of 24 KREDs are derived from the short-chain dehydrogenase of *Lactobacillus kefiri* (LKADH), this enzyme was also tried under these conditions, showing a conversion for this ketone that varied from 80% to >99% in both DES at 50% and 80% w/w. Hence, ChCl:Gly and ChCl:Sorb mixtures were used as co-solvents in the reduction of different methyl and ethyl ketones. KRED-P2-C11 was an active enzyme in the presence of 80% w/w DES giving excellent conversion results for all tested ketones except for 1-(naphthalen-1-yl)propan-1-one. In some cases, the stereoselectivity was improved by increasing DES percentage. For instance, KRED-P2-C11 enhanced significantly the *ee* from 78% to >99% for 1-phenylpropan-1-ol and it increased the *ee* from 54% to >99% for 4-phenylbutan-2-ol when comparing the reaction in plain aqueous buffer. They also explored the stability of KRED-P2-C11 in DES-buffer media at different temperatures. For this, the researchers performed the reduction of propiophenone with ChCl:Gly-buffer 80:20 (w/w) at 30 °C, 40 °C, and 50 °C. While the temperature was increased up to 40 °C, the bioreduction occurred more rapidly than at 30 °C, at 50 °C the enzyme partially lost its activity.

As a final goal, the researchers tried to test whether the excellent activity of KRED in the reduction of ketones in the neoteric solvent could be used in a one pot process combining a metal-catalyzed isomerization of different allylic alcohols with the asymmetric KRED-mediated reduction of the obtained ketones (Table 3). Firstly, the team focused on a sequential one-pot two-step methodology (entries 1–8). The metal-catalyzed isomerization was investigated in ChCl:Gly DES-buffer 50:50 w/w mixtures at 50 °C using 5 mol% of a ruthenium complex as a catalyst. When the isomerization was complete, the KRED and NADP^+^ were added without isolating the intermediate ketone. Then the mixture was stirred for 24 h at 30 °C. Different secondary alcohols were obtained with excellent to quantitative conversions and high to excellent isolated yields (60–95%) and *ee* values (93–99%). These results suggested that the KREDs were compatible with the reaction medium which came from the metal-catalyzed step, and that the impact of the Ru(IV) catalyst on the enzymatic performance was negligible. KRED enzymes got partially deactivated when the metal-catalyzed isomerization ran in a concurrent manner coupled with the bioreduction process in pure aqueous buffer, thus leading to low conversions into the allylic alcohols. However, when a mixture of DES-buffer 80:20 (w/w) was applied (entries 9–13) much better results were achieved. For instance, when 1-phenylprop-2-en-1-ol (**11a**) was incubated at 40 °C and 250 rpm in this medium containing both catalysts, after 24 h the substrate was converted to (*R*)-1-phenylpropan-1-ol (**12a**) with 90% conversion and >99% *ee*. Also, other racemic allylic alcohols (**11b**‒**d**) were successfully transformed to the enantiopure saturated (*R*)-alcohols **12b**‒**d** with good to excellent conversions (68–96%) [40].

### 2.3. Deep Eutectic Solvents and Reductases

**Scheme 4:**
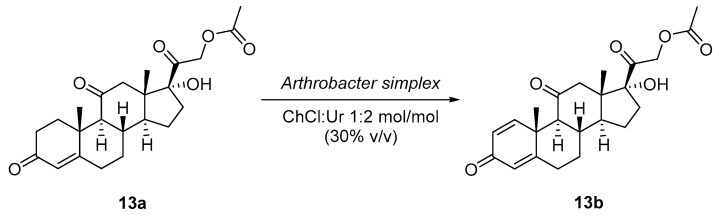
Bioconversion of cortisone acetate (**13a**) to prednisone acetate (**13b**) via ∆1,2-dehydrogenation mediated by *Arthrobacter simplex* whole cells in buffer/DES media.

Mao et al. [41] discovered that DES (ChCl:Ur 1:2 mol/mol) at 6% v/v improved the bioconversion of cortisone acetate (**13a**) to prednisone acetate (**13b**) via ∆1,2-dehydrogenation using immobilized whole cells of *Arthrobacter simplex* on calcium alginate (Scheme 4). At a substrate concentration of 5 g/L, the conversion was 93%, which was more efficient than the reaction without DES. The bacterial cells were immobilized and reused for 5 batches, still converting the substrate at >80%. Prednisone acetate is an anti-inflammatory agent, thus this study shows the relevance of the application of DES in the biocatalytic synthesis of anti-inflammatory and anti-allergy drugs. The authors also tested the toxicity of three types of DES (ChCl:Ur, ChCl:EG, and ChCl:Gly) at 4% v/v on *A. simplex* cells by measuring their influence on the bacterial membrane integrity. They found that ChCl:Ur decreased the membrane integrity to 39% and ChCl:EG decreased it into 51%. Normally cells consume glucose in non-toxic conditions, for this reason that group assessed the glucose uptake by the cells as a proof of toxicity and a decrease in this uptake was observed in cells that were dissolved in ChCl:EG and ChCl:Ur types of DES. Although the ChCl:Ur mixture was toxic to the bacterial cells, it was also able to permeabilize the cell membrane of the organisms and therefore increase the substrate solubility and binding to the enzyme(s). These results suggest that when designing a particular DES, the manufacturer has to keep the balance between permeabilizing the cell membrane and keeping the enzymatic activity, because at high concentrations it can be harmful as the mixture of components that make up the DES can be toxic.

In a subsequent work performed by the same research team, they assessed the effect of different cholinium salts on *Arthrobacter simplex* cells in the synthesis of **13b**. The authors found out that when the salts were used at high concentrations, they could have a toxic effect on the bacterial viability. Accordingly, they studied the effect of different DES (ChCl:Ur, ChCl:EG, and ChCl:Gly), of their isolated components, and the effect of the addition of both components in the reaction medium. Especially in the case of the ChCl:Ur solvent, it was observed that it enhanced the biotransformation conversion and the catalytic performance of the cells from *A. simplex*. Hence, the team confirmed that when this DES was used at low concentrations (30% v/v), it was not toxic to the bacteria. However, when it was used at higher concentrations (60% v/v), it had a poisonous effect on the cells. In another set of experiments, the effect of DES and their components on the membrane integrity of *A. simplex* cells was studied. Viability assays were carried out and the researchers concluded that the cell membrane of *A. simplex* in the presence of cholinium chloride was less stable compared to its presence with the different HBDs. The group also found out that the ChCl:Ur mixture was a good co-solvent in the steroid dehydrogenation reaction, and that the initial reaction rate for this reaction was higher compared to the ones that were performed in the DES individual components [42].

### 2.4. Deep Eutectic Solvents and Catalases

A study conducted by Harifi-Mood et al., showed the effect of two DES, namely reline (ChCl:Ur 1:2 mol/mol) and glyceline (ChCl:Gly 1:2 mol/mol), on the function and structure of bovine liver catalase (BLC). This enzyme is responsible of the decomposition of hydrogen peroxide into water and oxygen. At the 100 mM glyceline solution, the affinity of the substrate to the enzyme was enhanced. However, this was not observed when the enzyme was used in the reline solution due to the changes in the secondary structure of the protein. Also, at this concentration the enzyme stayed 70% active in the glyceline solution and it showed 80% activity in the reline solution compared to the plain aqueous solution. On the other hand, BLC showed nearly 40% activity in the ChCl solution (1.5 M) because of the chaotropic nature of the cholinium cation, which stops the reducing activity of the catalase in the concentrated solutions of this salt. On the other hand, this effect was not seen in DES mixtures. BLC is known as an acidic protein and several negative charges can be found on the surface of this tetrameric protein. For this reason, the structure of that enzyme can change via the neutralization of its charges. At low concentrations of DES, the BLC activity was not affected by the overall molar ratio of the choline cations which were present in the DES. Urea protected the catalase activity by stabilizing the tetrameric conformation of the biocatalyst [43].

The general reaction of a catalase occurs in two steps. In the first one the hydrogen peroxide oxidizes the heme iron of the enzyme forming an oxyferryl group with a π-cationic porphyrin radical, called Cpd I, releasing water. In the second step, Cpd I reacts with another molecule of hydrogen peroxide leading to the enzyme to its resting state releasing a second molecule of water and one of oxygen. When the hydrogen peroxide concentrations are low and when there is a one-electron donor, Cpd I can directly undergo to another intermediate (Cpd II). Cpd II has an important function in the reversible deactivation of catalase by its substrate. When catalase is exposed to hydrogen peroxide which is generated at a constant rate, the enzyme can become inactive because it reaches a steady state. Cpd II formation can be measured at 435 nm spectrophotometrically. Thus, the formation of this intermediate during the BLC reaction in the presence of glyceline, reline, and choline chloride in aqueous buffer solutions was investigated at 435 nm. The production of Cpd II in the reline solution was faster than in the glyceline solution, therefore, reline medium played a more important inhibitory role compared to the glyceline solution. Also, Cpd II concentration in the choline chloride solution was higher than its concentration in the solutions that contained DES. The synergistic effect of both choline cations and HBDs in reline and glyceline solutions decreased the formation of this undesired intermediate. In addition, the authors also conducted a structural study of the enzyme by using fluorescence and circular dichroism (CD). They found less structural changes in the presence of reline and glyceline solutions compared to the choline chloride solution. There was a decrease in the helical part of the enzyme in the presence of ChCl or reline, demonstrating the interference with the protein and the disruption of the hydrogen bonds of the protein in the alpha helix regions. The disruption led to the decrease of the substrate affinity to the protein. The team also found that the glyceline solution gave a more stable secondary structure of the BLC by increasing the contents of the α-helixes and the β-sheets.

## 3. Recent Biotechnological Applications

In the last years, DES have found potential applications in different biotechnological fields. Herein we just want to briefly mention a few recent interesting uses in this field. In the last decades, biofuels are produced in a less harmful way to the environment, but they are usually obtained at a low purity. In this context, Niawanti and co-workers purified crude biodiesel using DES. As these solvents have high polarities, they were able to separate biodiesel from its impurities like glycerol, water, free fatty acids (FFA), monoglycerides (MG), diglycerides (DG) and triglycerides (TG). This study also confirmed that longer extraction times augmented the purity degree of the biodiesel [44].

Capturing post-combustion carbon with aqueous amines is very efficient because of the high absorption capacity of carbon dioxide and its high selectivity for that latter compared to nitrogen. The solubility of carbon dioxide in the presence of primary, secondary, and tertiary amines varies depending on the reaction equilibrium. The research group led by Alnashef et al. [45], have developed a method to solubilize and capture carbon dioxide using three different DES containing amino alcohols. The first one was composed of ChCl and ethanolamine (ChCl:MEA). The second one was made of ChCl and diethanolamine (ChCl:DEA). The constituents of the third one were ChCl and diethanolmethylamine (ChCl:MDEA). Each solvent was obtained at different molar ratios (1:6, 1:8 and 1:10, respectively). It was found that these amine-based DES had an absorption capacity higher than the ones corresponding to the aqueous amino alcohol solutions and other regular DES. Also, it was discovered that DES composed of ChCl:MEA absorbed the highest quantity of carbon dioxide, and the one that absorbed the least was the one composed of ChCl:MDEA. Hence, the ChCl:MEA mixture was able to solubilize carbon dioxide at 265% compared to the aqueous solution of the amine (at 30% w/v) since it was able to absorb carbon dioxide both physically and chemically. However, the DES composed of ChCl:MDEA absorbed poorly carbon dioxide (16% regarding the aqueous solution of the amine at 30% w/v).

A study developed by Durand et al. has found that when antioxidants were formulated in NADES, they were more efficient at inhibiting ROS than in aqueous medium. Researchers looked for a method that assayed in vivo the activity of the antioxidants derived from phenolic compounds. For this, a conjugated autooxidizable triene assay (CAT) was developed. Eleven antioxidants were evaluated using the CAT assay and fibroblast cell lines to identify molecules with high ROS inhibiting activities. The antioxidants that showed less activity were 3,4-dihydro-6-hydroxy-7-methoxy-2,2-dimethyl-1(2*H*)-benzopyran palmitate (CR-6 palmitate), α-tocopherol acetate and capsiate due to the fact that these antioxidants did not have enough affinity to cell membranes. On the other hand, bis(2-ethylhexyl) 2-(4-hydroxy-3,5-dimethoxybenzyl)malonate (Bis-EHBm), hydroxytyrosol acetate and hydroxytyrosol were the most efficient at inhibiting ROS in cell assays. Finally, the research team confirmed that the use of different antioxidants in a NADES formulation (ChCl:propane-1,2-diol 1:1 mol/mol) improved their ROS inhibition activity. In some cases, it was improved up to 360% regarding the aqueous medium. The authors claimed that the NADES may facilitate cellular membrane permeation and transport more rapidly the antioxidant to the location where it is active [46]. All the previous studies have shown the importance and the various applications of DES in different biotechnological fields.

## 4. Conclusions and Perspective

This review shows some major advantages of DES in oxidoreductase-mediated transformations. It focuses on the different uses of DES and their effects on living cells. In addition, the properties of DES were clearly affected by the molar ratios of HBA and HBD. DES were also proved to be useful in separating compounds that are important in the pharmaceutical industry. Some studies throughout this review used different DES to study the effect in the activity and stereoselectivity by using whole cells. Overall, DES can improve in some cases the productivity of redox reactions and can be successfully applied for various biotechnological processes. This review also serves as a guide for researches interested in this type of medium because it highlights the latest applications of DES using different enzymes and microorganisms. While up to now mainly whole cell-mediated transformations have been used in redox biocatalytic reactions in the presence of DES, it is clear that in the next years the application of isolated enzymes in these solvents will be highly appealing. The introduction of DES in cascade or sequential multicatalytic protocols will also be developed due to the relevance that these methods are acquiring in the last years. While until now the best DES mixtures are the ones composed of choline chloride and glycerol, it is expected that new applications will be found for, e.g., the cofactor recycling, which is necessary in oxidoreductase-mediated reactions [47].

## Figures and Tables

**Table 1:** The effect of deep eutectic solvents (DES) on different organisms.

Organism	Effect of DES	Reference
Bacteria	DES composed of choline chloride (ChCl) was not toxic	[7,8]
Marine organisms	*Hydra sinensis*: ChCl based DES led to the decomposition of their bodies Brine shrimp: DES was lethal and toxic	[8]
Human cells (cancer cells)	Disrupts the cell membranes and decreases the proliferation of cancer cells	[9]
Plants	Decreases chlorophyll and causes disequilibrium in the homeostasis and cell walls of plant cells	[10]
Mice	Urea type of DES causes death at high levels	[11]

**Table 2:**
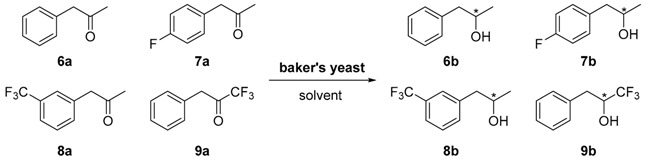
Selective reduction of ketones **6-9a** to their corresponding enantioenriched alcohols **6-9b** using baker’s yeast in different solvent conditions [38].

Substrate	Solvent	Conversion (%)	*ee* (%)
**6a** ^(a)^	water	88	96 (*S*)
**6a** ^(a)^	DES A (60% w/w) in water ^(c)^	31	78 (*S*)
**6a** ^(a)^	DES B (50% w/w) in water ^(d)^	88	88 (*S*)
**6a** ^(a)^	DES B (90% w/w) in water ^(d)^	36	96 (*R*)
**7a** ^(b)^	water	82	94 (*S*)
**7a** ^(b)^	DES B (90% w/w) in water ^(d)^	10	90 (*R*)
**8a** ^(b)^	water	90	98 (*S*)
**8a** ^(b)^	DES B (90% w/w) in water ^(d)^	15	40 (*R*)
**9a** ^(b)^	water	>95	2 (*S*)
**9a** ^(b)^	DES B (90% w/w) in water ^(d)^	12	24 (*R*)

^(a)^ Reaction conditions: ketone **6a** (1.5 mM), baker’s yeast (230 mg/mL) at 37 °C. ^(b)^ Reaction conditions: ketones **7**-**9a** (1.5 mM), baker’s yeast (200 mg/mL) at 37 °C. ^(c)^ DES A: ChCl:D-fructose (3:2 w/w). ^(d)^ DES B: ChCl:Gly (1:2 mol/mol).

**Table 3:**
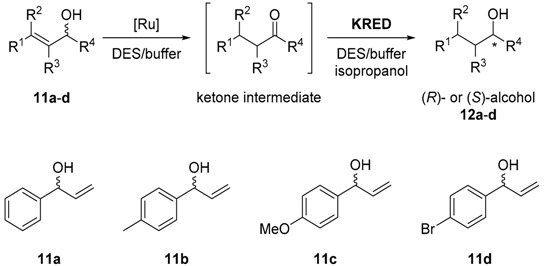
The conversion and enantiomeric excess results of a sequential process reaction combining the use of a ruthenium-catalyzed isomerization and a bioreduction in the presence of DES [40].

Entry	Substrate	Product	Solvent	ADH	Conversion (%)^(c)^	*ee* (%)
1 ^(a)^	**11a**	**12a**	ChCl:Gly (1:2 mol/mol, 50% w/w)	P1-A04	>99 (95)	>99 (*R*)
2 ^(a)^	**11a**	**12a**	ChCl:Gly (1:2 mol/mol, 50% w/w)	*L. kefiri*	>99 (92)	>99 (*R*)
3 ^(a)^	**11b**	**12b**	ChCl:Gly (1:2 mol/mol, 50% w/w)	P2-H07	95 (90)	>99 (*R*)
4 ^(a)^	**11b**	**12b**	ChCl:Gly (1:2 mol/mol, 50% w/w)	*L. kefiri*	70 (64)	93 (*R*)
5 ^(a)^	**11c**	**12c**	ChCl:Gly (1:2 mol/mol, 50% w/w)	P3-H12	95 (90)	>99 (*S*)
6 ^(a)^	**11c**	**12c**	ChCl:Gly (1:2 mol/mol, 50% w/w)	*L. kefiri*	65 (60)	93 (*R*)
7 ^(a)^	**11d**	**12d**	ChCl:Gly (1:2 mol/mol, 50% w/w)	P2-H07	94 (90)	>99 (*R*)
8 ^(a)^	**11d**	**12d**	ChCl:Gly (1:2 mol/mol, 50% w/w)	*L. kefiri*	80 (72)	98 (*R*)
9 ^(b)^	**11a**	**12a**	ChCl:Gly (1:2 mol/mol, 80% w/w)	P2-C11	90	>99 (*R*)
10 ^(b)^	**11b**	**12b**	ChCl:Gly (1:2 mol/mol, 80% w/w)	P2-C11	70	>99 (*R*)
11 ^(b)^	**11c**	**12c**	ChCl:Gly (1:2 mol/mol, 80% w/w)	P2-C11	68	>99 (*R*)
12 ^(b)^	**11d**	**12d**	ChCl:Gly (1:2 mol/mol, 80% w/w)	P2-C11	96	>99 (*R*)
13 ^(b)^	**11a**	**12a**	ChCl:Gly (1:2 mol/mol, 80% w/w)	*L. kefiri*	21	>99 (*R*)

^(a)^ Reaction performed in a sequential mode. ^(b)^ Reaction performed in a concurrent manner. ^(c)^ Isolated yields appear in brackets.
